# Supplementary material for: Talking about Health: A Topic Analysis of Narratives from Individuals with Schizophrenia and Other Serious Mental Illnesses
Source: Behav Sci (Basel). 2022 Aug 13;12(8):286. doi: 10.3390/bs12080286 (PMC9405157; doi:10.3390/bs12080286)
Supplement: Supplementary file 1 [file behavsci-12-00286-s001.zip › behavsci-1793193-supplementary.pdf]

Supplementary Table S1. Frequency counts for different groups and top 10 desc
text were assigned nicknames based on qualitative ins

Topic	Nickname	Control	Schizophrenia	SMI Other	Black	Other	White
-1	-	60	278	83	134	7	209
0*	food	33	41	30	36	2	51
1*	hobbies	28	38	32	34	2	44
2	peace	26	33	24	23	3	40
3	routine	0	68	2	7	0	36
4	memories	7	58	5	22	0	26
5	home	0	28	26	13	2	22
6	life	0	28	21	22	0	17
7	home	21	13	14	6	2	27
8	happy	12	18	5	14	0	22
9	sad	6	17	8	4	0	17
10*	scared	2	26	2	1	0	28
11	-	1	27	0	10	1	8
12	angry	14	8	4	6	0	14
13*	religion	3	8	14	3	1	10
14	-	0	17	8	5	1	11
15	work	0	21	1	1	0	10
16	-	0	21	0	8	1	6
17	-	2	12	4	3	0	11
18	-	6	7	2	8	0	6
19	-	2	7	5	4	0	8
20	-	0	13	0	5	0	10
21	-	3	8	2	2	0	7
22	-	3	7	3	8	0	1
23	-	2	4	4	3	0	7
24	-	0	9	0	4	0	7
25	-	1	5	3	0	0	5
26	-	1	7	1	2	1	5
27	-	1	2	5	0	0	7
28	mind	0	2	6	1	0	8
29	-	1	7	0	5	0	2
30	-	0	8	0	5	0	1
31	-	2	4	2	2	0	5
32	-	5	3	0	2	0	3
33*	mental	0	4	3	4	0	2
34	-	0	7	0	2	2	3
35	-	0	3	4	2	0	4
36	-	0	5	1	4	0	4
37*	time	0	6	0	4	0	3
38	-	2	4	0	3	0	4
39	-	0	3	3	3	0	4
40	-	1	2	2	2	1	2
41	-	2	2	1	0	0	3

criptive words for each topic. An asterisk (*) is placed next to health-related topics. Topics mentioned in the main peciton of the top 10 words. N.B. Topic "-1" is a "catch-all topic" and should be ignored.

Top 10 Words
know, just, got, did, really, going, time, lot, good, kind
food, eat, cook, love, chicken, cooking, good, eating, favorite, lot
hobbies, hobby, play, watch, love, just, read, lot, music, favorite
peace, feel, just, relaxed, outside, calm, felt, really, peaceful, good
usually, work, day, know, just, home, 0, time, ah, watch
know, fun, just, good, went, lot, used, memories, time, remember
know, people, think, ah, just, person, things, lot, time, kind
know, ah, just, got, people, want, life, going, yes, good
room, live, bedroom, living, kitchen, house, living room, nice, roommate, tv
happy, really, time, just, know, good, got, used, did, real
sad, felt, low, energy, feel, low energy, feel sad, passed, times, away
know, did, bad, snake, crap, got, time, going, sad, went
know, just, got, yeah, going, really, lot, time, want, stuff
furious, angry, youknow, really, disgusted, just, person, mad, upset, people
church, know, god, things, just, wood, bible, say, people, think
years, just, lot, work, sss, know, kind, really, time, going
know, really, going, new, just, kind, got, trying, time, work
know, just, bad, really, did, time, people, things, kind, think
happy, really happy, best, really, special, times, feeling best, really special, feel, feeling
died, just, funeral, hard, away, did, going, time, know, passed away
live, woodbury, lll, river oaks, oaks, good, living, place, river, love
stressful, know, did, stress, time, lot, bad, got, going, died
fish, fishing, fun, just, know, ah, really, mean, kind, boat
dog, cat, youknow, rat, vet, day, rid, growl, pet, imean
just, sick, felt, hospital, kind, bad, rrlike, stuff, blood, know
3E+36, 393c 3e36, 393c, just, know, let, got, kind, stuff, things
okay, ring ring, got, ring, fight, ring ring ring, school, angry, classroom, funny
know, want, lot, month, feel, things, going, people, just, think
scared, disgusted, feel, furious, really scared, times, times really, smoke, really, times feel
ah, ged, know, going, magic, medication, years, church, lot, work
good, did, school, good time, got, time, just, know, team, went
got, yeah, did, going, went, oh, know, account, pretty, tim
ugh, died, sad, hospital, death, believe, loved ones, dad, divorced, love
bad, energy, energized, felt, felt bad, energetic, feeling, times, times really, feel
live, living, mental, absolutely, grace, place, people, group home, home, transitional
know, did, okay, yeah, people, just, things, bad, got, time
just, did, said, know, want, mom, cousin, feel, kids, got
yeah, nick, ex, kevin, ex husband, got, guy, yeah yeah, yeah oh, oh
work, just, really, time, ah, job, actually, month, kids, ccc
happy, feel good, good, feel, kept, try stay, popeyes, dependent, pizza, came
good, times, rode, coaster, roller coaster, remember, time, memorable, roller, memories
ah, baby, did, american, got, know, said, figured, really, looked
came, told, came told, stuff house, attorney, husband came told, appointment attorney, appointment attorney said,

told coming, attorney said
